# Xanthohumol promotes cognitive function-associated neuroprotein changes in two mouse models of Alzheimer’s disease

**DOI:** 10.1016/j.ibneur.2026.10.013

**Published:** 2026-10-23

**Authors:** Wei Liu, Benhong Xu, Chen Yang, Guowei Li, Xifei Yang

**Affiliations:** a Shenzhen Center for Disease Control and Prevention, Shenzhen 518055, China; b Shenzhen Key Laboratory of Modern Toxicology, Shenzhen Medical Key Discipline of Health Toxicology (2020-2024), Shenzhen Center for Disease Control and Prevention, Shenzhen 518055, China

**Keywords:** Alzheimer’s disease, Xanthohumol, Cognitive impairment, Proteomics, Tandem mass tags, Mouse

## Abstract

Alzheimer's disease (AD) is a neurodegenerative disorder that progressively impairs cognitive function for which there is no effective treatment. Natural dietary compounds such as xanthohumol (Xn) potentially offer a new therapeutic approach in AD. We aim to employ quantitative proteomic techniques to investigate the effects of Xn on cognitive impairment in two established murine models of AD. Treatment with 0.5 mg/kg Xn significantly improved the ability of stimulating learning and memory and cognitive function of 3 ×Tg AD mice while 5 mg/kg Xn ameliorated cognitive decline in APP/PS1 mice. We identified two common differential proteins and 12 pathways in the hippocampi of 3 ×Tg AD mice and 70 common differential proteins and 22 pathways in APP/PS1 mice. Moreover, Nine common signaling pathways mainly involved in energy metabolism, neurodegenerative disease, cardiovascular disease and cancer. So multi high-throughput proteomic analyses of hippocampus add to the scientific basis for development of effective drugs for AD, one candidate of which is Xn.

## 1 Introduction

Alzheimer’s disease (AD) is a chronic progressive neurodegenerative disease with an insidious onset and with clinical presentation most commonly in old age. AD may account for 60–70% of cases of dementia and is one of the top ten causes of death (Alzheimer's Association Report, 2023). AD brings a huge physical, psychological, social and economic impact not only for the patients themselves but also for their caregivers, families and the entire society (Cavaco et al., 2025, Ren et al., 2022). The etiology and pathogenesis of AD are not understood, and there is currently no effective treatment to slow or stop the downhill clinical course of the brain disease. The present study employs murine models of AD to test the effect of daily systemic treatment with xanthohumol (Xn).

Hops (*Humulus lupulus L.*) is a kind of medicinal and edible homologous plant with a long history, and is one of the main raw materials in brewing beer. Xn is a component of the female flower extract from hops that has been tested for its biological effects in various health disorders, including metabolic syndrome (Gómez-Zorita et al., 2024), osteoarthritis (Sun et al., 2023), diabetic nephropathy (Zhao et al., 2023), and cancer (Li et al., 2023). In experimental studies, Xn significantly reduced memory impairment of APP/PS1 mice (Sun et al., 2021), inflammation and apoptosis of aged brains (Zhang et al., 2025), ameliorated ApoE isoform-dependent effects on cognitive performance (Kundu et al., 2022) and inhibited tau protein aggregation and disaggregated tau fibrils (Zhang et al., 2019). Importantly, two Xn derivatives, Xn-3 and Xn-9, were found to be blood-brain barrier permeable and neuroprotective (Wang et al., 2019). In our previous study (Huang et al., 2018), Xn significantly suppressed Aβ production and tau hyperphosphorylation in N2a/APP cells via APP processing and the GSK-3β pathway. Moreover, Xn reduced cognitive dysfunction and significantly regulated the diversity of intestinal microflora in young and old APP/PS1 mice (Liu et al., 2022). These data suggested Xn may be a promising candidate for the treatment of AD. However, the specific protective mechanism of Xn in animal models of AD remains unclear.

Proteomic techniques play a key role in discovering disease biomarkers and in understanding molecular mechanisms of drug action (Hanash, 2003). Both labeling of peptides and label-free approaches combined with liquid chromatography tandem mass spectrometry (LC-MS/MS) have been applied to various diseases (Cao and Yu, 2022, Gajula et al., 2023). Two-dimensional difference in-gel electrophoresis (2D-DIGE) is a classical and widespread technique in proteomics for the identification of biomarkers (Sivagnanam et al., 2022). Tandem Mass Tag (TMT) technology, developed by Thermo Fisher Scientific Inc. in the USA, is a relatively new protein quantitation technique, based on in vitro isotope labeling, which has been widely used in proteomics research in recent years (Adegboye et al., 2025, Wang et al., 2024). These techniques are employed in the present study to illuminate mechanisms underlying the effects of Xn treatment in slowing cognitive decline in 3 ×Tg AD mice and APP/PS1 mice.

## 2 Materials and methods

### 2.1 Animals and treatments

The study was approved by the ethics committee of Shenzhen Center for Disease Control and Prevention (No. 2019034, No. 2022019). Animal treatment and care were conducted in accordance with the ethics principles of Declaration of Helsinki. Two murine models were used: *3 ×Tg* AD mice and *APP/PS1* AD mice (Supplementary Figure 1). Combined with the biological characteristics of Xn (such as poor bioavailability, but an ingredient of beer) and the existing references (Legette et al., 2012, Stevens and Page, 2004, Yen et al., 2012), we first observed the improvement of cognitive function of *3 ×Tg* AD mice by intraperitoneal injection of Xn. We then analyzed the promoting effect of Xn oral administration on cognitive function of *APP/PS1* AD mice. Each group of mice comprised 10–14 animals, half male and half female.

Approximately 7-month-old *3 ×Tg* AD mice (B6129SF2/J background) and age- and sex-matched control littermates were used in this study. Xn, (Cat# 6754–58–1, stated purity ≥97.0%) purchased from Aladdin Biochemical Technology Co. (Shanghai, China), was dissolved in a solvent mixture (vehicle) of ethanol (Cat# 64–17–5), polypropylene castor oil (Cat# 61791–12–6) and saline (Cat# 7647–14–5, 1:1:4, respectively). Mice were divided into six groups (10 animals per group): (1) *3 ×Tg* AD mice (model group) and wild-type (WT) mice (control group) treated with 0.1 mL/10 g body weight of solvent mixture, and an equivalent volume was used to treat (2) WT mice with 0.1 mg/kg Xn (low-dose control group) or 0.5 mg/kg Xn (high-dose control group) and (3) *3 ×Tg* AD mice with 0.1 mg/kg Xn (low-dose test group) or 0.5 mg/kg Xn (high-dose test group). The dosage and administration of Xn were selected based on a previous report (Yen et al., 2012). Xn and vehicle were administered daily by intraperitoneal injection for six weeks.

*APP/PS1* AD mice with an average age of six months and age-matched C57 mice with the same genetic background were used in the second murine study. Mice were divided into four groups of 11–14 mice per group: WT mice (control group), WT mice treated with 5 mg/kg Xn, *APP/PS1* AD mice (model group), *APP/PS1* AD mice with 5 mg/kg Xn (test group). Animals were treated with Xn or corn oil (0.1 mL/10 g body weight) by oral gavage every other day for three months. The oral dosage and administration of Xn was based on a previous report (Legette et al., 2012).

### 2.2 Behavioral tests

The *3 ×Tg* AD mice with or without Xn treatment performed the step-down passive avoidance (SPA) test and Morris water maze (MWM) test, while the *APP/PS1* AD mice only performed the MWM test.

#### 2.2.1 SPA test

The SPA test was used to evaluate learning and memory in mice (Kameyama et al., 1986), and performed following our previous report (Wang et al., 2019). Briefly, the mice was placed at the bottom floor and received electric shock (constant voltage 35 V, shock intensity 0.5 mA), and the normal response was to jump on the platform to avoid the harmful stimulus. After 24 h during the 5-min test period, the mice was placed on the platform and the electric shock was also delivered in the floor. The time required for the animal to jump off the platform for the first time namely step down latency (SDL) and the number of jumps off the platform namely error times (ET) were recorded. The shorter the SDL, the more ET indicated that the memory impairment was more obvious.

#### 2.2.2 MWM test

To evaluate spatial learning and memory of mice, the MWM test was used in accord with the published method (Vorhees and Williams, 2006) and our previous work (Liu et al., 2022). Briefly, during the 5-days training period, animals were placed in the pool from the corresponding positions of the four quadrants, and then observed for 60 s. If the mice found the platform and stayed on the platform for 3 s, the observation was terminated, and the time from entering the water to finding the platform was recorded as the escape incubation period. The mean incubation period of 4 times per day was calculated to evaluate spatial learning ability of animals. On the 7th day, the platform was removed. The observation time was 2 min. The system automatically recorded the time when the mice reached the original platform for the first time (spatial probe time), the times of crossing the platform, the mouse swimming track and other parameters.

### 2.3 Separation and collection of hippocampus tissue

After the behavioral experiments, the mice were anesthetized and the brain was removed and placed in pre-cooled 0.9% normal saline. The hippocampal tissue was immediately separated on ice and quickly stored at -80℃.

### 2.4 Hippocampus proteomics study in *3 ×Tg* AD mice by two-dimensional difference gel electrophoresis (2D-DIGE) technique

Hippocampus proteomics analysis was performed by 2D-DIGE coupled with MALDI-TOF-MS/MS as described with the reported method (May et al., 2021) and our previous work (Chen et al., 2018, Zhang et al., 2021). Briefly, hippocampal protein was firstly extracted, purified by acetone (Cat# 67–64–1) precipitation and quantified according to the instructions of 2-D Quant kit (Cat# 80–6483–56, GE Healthcare, USA). Samples were labeled with Cy3 and Cy5 fluorescent dye solution. Labeled samples were incubated, separated by sodium dodecyl sulfate-polyacrylamide gel electrophoresis (SDS-PAGE). The Typhoon multi-functional laser scanning imaging system was used to collect images, and DeCyder V6.5 software was used to determine protein points with significant differences (*P* < 0.05).

The extracted protein glue spots were dehydrated with anhydrous acetonitrile (Cat# 75–05–89), and treated overnight with 5 µl 20 µg /mL pancreatic enzyme (Cat# 68920–15–0) and 15 µl 20 mM ammonium bicarbonate (Cat# 10361–29–2) at 37℃. After enzyme digestion, protein samples (1 µL) were identified by MALDI-TOF MS with the following parameters: 355 nm nitrogen laser; delay extraction time of 300 ns; first ion source of 25.00 kV, second ion source of 23.90 kV; collection range of 1–20 kDa. MASCOT software and UniProt *Mus musculus* database were combined to conduct a protein search database and identify the differential proteins.

### 2.5 Hippocampus proteomics study in *APP/PS1* AD mice by tandem mass tags (TMT) technique

Hippocampus proteomics analysis was performed by nano liquid chromatography (Nano-LC) coupled with TMT labeling as described with the reported method (Pagel et al., 2021, Shen et al., 2018, Zhang and Elias, 2017) and our previous work (Xu et al., 2022). Briefly, hippocampal tissue was first sonicated, ice lysed, and centrifuged at 14000 RPM, 4℃ for 15 min. The total protein in the supernatant was quantified by bicinchoninic acid (BCA, Cat# 214–990–9) method. 400 μg protein from each sample was enzymolized. The supernatant was then desalted, rotated and dried in vacuum for further TMT labeling. 200 mM triethylamine bicarbonate (TEAB, Cat# 384347–06–2), 41 μL TMT Reagent (Cat# 90067) and 8 μL 5% hydroxylamine (Cat# 7803–49–8) were added to each sample. Each solution was mixed in sequence, and then adjusted pH 1–2. The mixed sample was desalted, rotated and dried, and redissolved by adding 100 μL 0.1% formic acid (FA, Cat# 64–18–6), and centrifuge at 12,000 RPM for 3 min. The supernatant was transferred to a new tube for peptide separation.

The TMT-labeled peptides were first separated by XBridge® peptide BEH C18 column (300 Å, 4.6 × 250 mm, 5 μm, Waters, Milford, Massachusetts, USA) for HPLC separation (UltiMate 3000 UHPLC, Thermo Scientific, Waltham, MA, USA). The polypeptides were then separated using a tandem-linked pretreatment column and an analysis column, and further analyzed by LC-MS/MS to identify peptides and quantify reporter ion relative abundance (Q-exactive mass spectrometry, ThermoScientific, MA, USA). The identified peptides were then analyzed by Proteome Discoverer (version 2.1.0.81, Thermo Scientific, Waltham, MA, USA).

### 2.6 Bio-informatics analysis

Heat maps and volcano maps were performed using the OmicStudio tools (https://www.omicstudio.cn/tool). Panther online software (http://www.pantherdb.org/), was used for function enrichment analysis of hippocampal differential expressed proteins, according to the biological process, molecular functions, components and protein pathways. Gene Ontology (GO) analysis and Kyoto Encyclopedia of Genes and Genomes (KEGG) pathway analysis was performed by the Database for Annotation, Visualization and Integrated Discovery (DAVID) Bioinformatics Resources (Oct. 2023 update, https://david.ncifcrf.gov/tools.jsp; (Sherman et al., 2022)).

### 2.7 Statistical analysis

SPSS version 25.0 was used for data analysis. Two-way ANOVA was used to compare the mean between groups followed by the Tukey’s multiple comparisons test, which was used for both behavioral tests. Repeated measures ANOVA was used in the MWM test. Graphs were drawn via Graphpad Prism Software (version 8, La Jolla, CA). The Mann Whitney Wilcoxon test was applied to measure the significance of each peak in the different groups of proteomic analysis, with results adjusted for multiple testing using false discovery rates (FDR) correction. Two-sided *P* values < 0.05 were considered statistically significant.

## 3 Results

### 3.1 Xn significantly relieved cognitive impairment of 3 ×Tg AD mice

SPA test results (Fig. 1) showed that, compared with WT mice, the SDL of *3 ×Tg* AD mice was significantly decreased and the ET was significantly increased, both consistent with memory impairment. Treatment with 0.5 mg/kg Xn significantly improved the ability of stimulating learning and memory of *3 ×Tg* AD mice (*t* = 144.5, *P* = 0.0072 for latency. *t* = -1.6, *P* = 0.0029 for error times). The MWM test (Fig. 2) showed that with the increase of training days, the time for WT and *3 ×Tg* AD mice to find the platform became shorter and shorter, but there was no statistical difference among the groups (*F*_*total*_=1.198, *P* = 0.2605). Compared with 3 ×Tg AD mice, Xn-treated AD mice had more concentrated movement routes in the target quadrant. Relative to the performance of Xn-untreated *3 ×Tg* AD mice, the number of times mice traversed the platform markedly increased (*t* = 3.2, *P* = 0.0023) and the time spent exploring the platform decreased significantly (*t* = -29.9, *P* = 0.014). The activity distance and time percentage spent in the target quadrant also increased significantly with Xn treatment. In sum, compared with WT mice, *3 ×Tg* AD mice had obvious learning and memory impairment that was significantly relieved by Xn treatment.

**Fig. 1:**
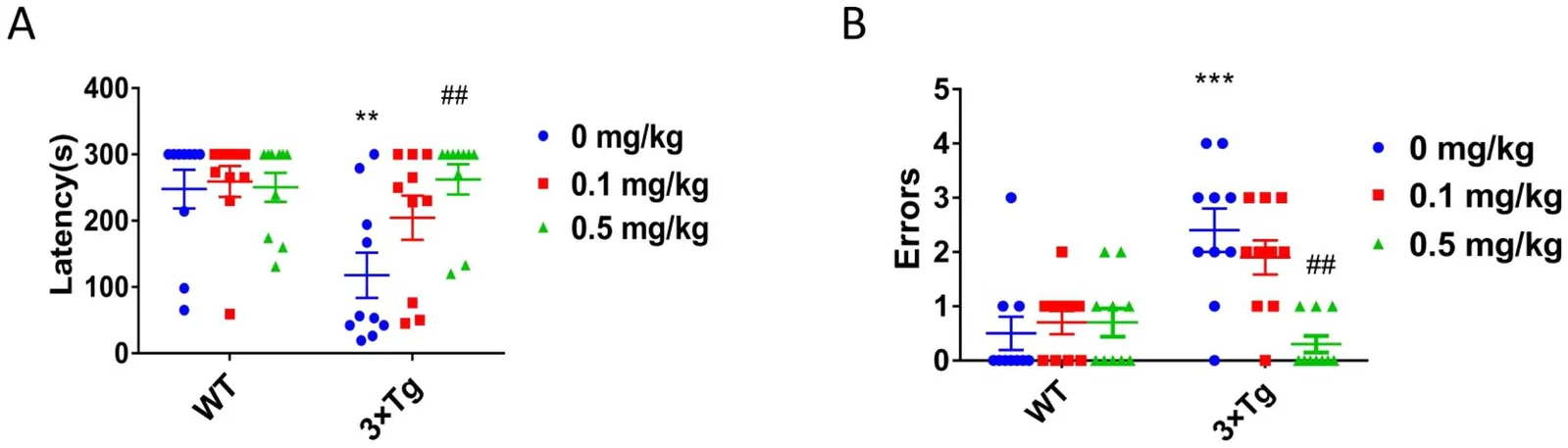
Xn treatment significantly improved the ability of stimulating learning and memory of 3 ×Tg AD mice. (A) the step down latency (SDL) for animals to jump off the platform for the first time; (B) the error times (ET) of mice to jump off the platform. n = 10 per group (five male and five female). ** *P* < 0.01 vs. WT, *** *P* < 0.001 vs. WT, # # *P* < 0.01 vs. 3 ×Tg AD mice. Error bar, standard error of mean (SEM).

**Fig. 2:**
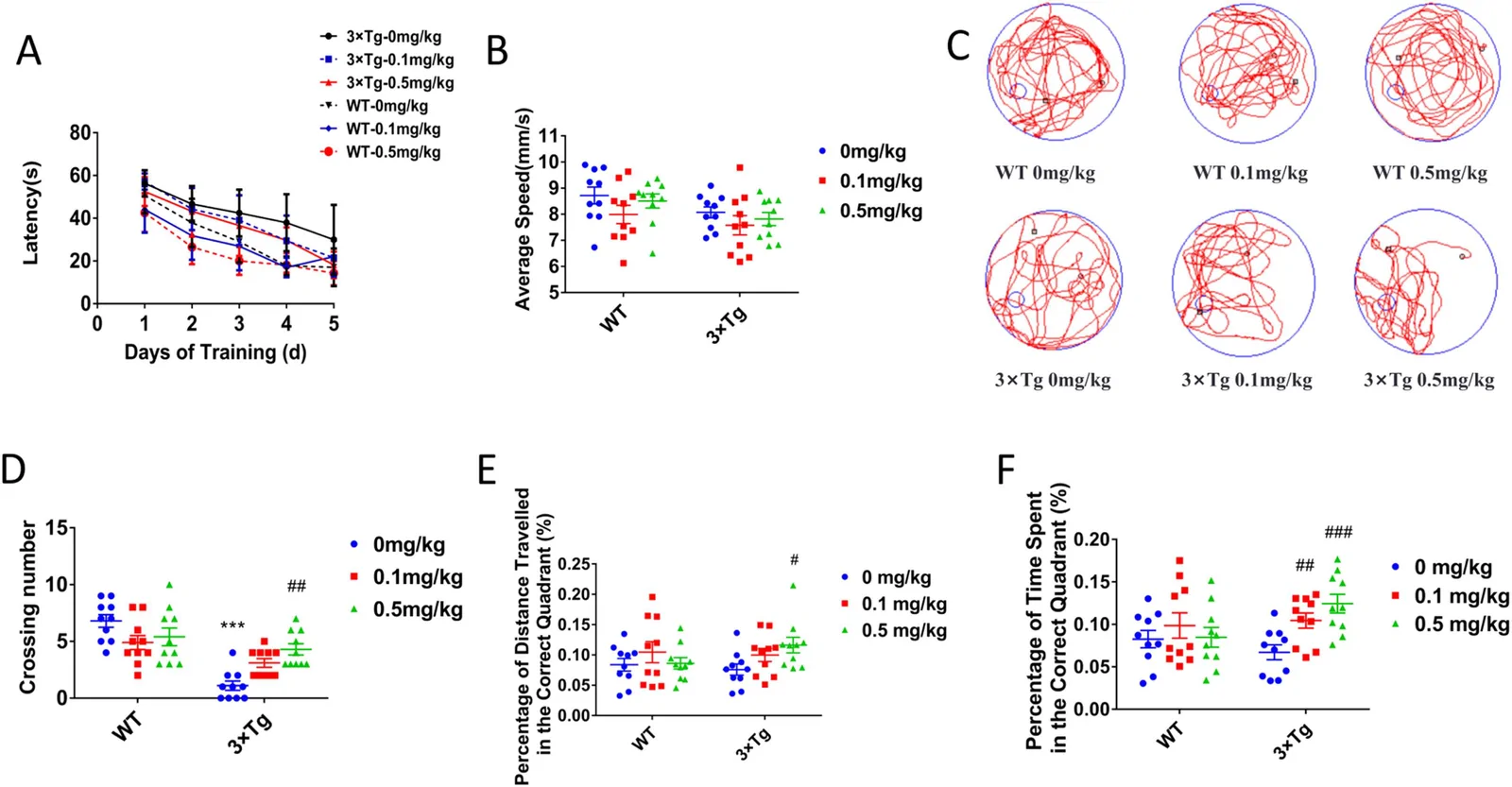
Xn treatment improved the learning and memory ability of 3 ×Tg AD mice. (A) escape latency of animals to find the platform for the first time during the platform navigation test; (B) average speed during the spatial probing test; (C) motion trajectory diagram; (D) number of times animals crossed the platform; (E) distance traveled in the target quadrant as a percentage of total distance traveled; (F) time spent in the target quadrant as a percentage of total time. n = 10 per group (five male and five female). *** *P* < 0.001 vs. WT, # *P* < 0.05 vs. 3 ×Tg AD mice, # # *P* < 0.01 vs. 3 ×Tg AD mice, # # # *P* < 0.001 vs. 3 ×Tg AD mice. Error bar, standard error of mean (SEM).

### 3.2 Xn ameliorated learning and memory in APP/PS1 mice

The MWM test was performed to evaluate learning and memory ability of *APP/PS1* mice vs. WT animals. Compared with untreated *APP/PS1* mice, Xn-treated animals showed better performance (Supplementary Figure 2). Over the 5-day training period, all mice took less and less time to find the hidden platform (*F*_*total*_=2.893, *P* = 0.0444), with the escape latency of Xn-treated and -untreated WT mice leading the way. Moreover, Xn-treated mice spent less time than the Xn-untreated mice, with female *APP/PS1* mice more obvious than male mice (*F*=5.122, *P* = 0.0022). Thus, Xn treatment enhanced the learning ability of both *APP/PS1* and WT animals. In the probe period, compared with the *APP/PS1* group, Xn-treated mice crossed more frequently within the platform area and spent more time and distance in the target quadrant (*t* = 8.681, *P* = 0.0478; *t* = 9.198, *P* = 0.0217). These results indicate that Xn treatment ameliorated cognitive decline in APP/PS1 mice.

### 3.3 Xn altered the expression levels of hippocampal proteins in 3 ×Tg AD mice

To investigate the effect of Xn on hippocampus proteins content of *3 ×Tg* AD mice, three groups with significant differences on their behavioral results were selected for protein analysis. The representative 2D-DIGE images of differential protein spots were identified by MALDI-TOF-MS/MS (Supplementary Figure 3). Compared with WT mice, a total of 30 hippocampal proteins were statistically significant in *3 ×Tg* AD mice, of which 19 were low-expressed and 11 were high-expressed (Supplementary Table 1). Compared with *3 ×Tg* AD control mice, a total of 27 proteins were statistically different after Xn treatment, including 11 proteins with low expression and 16 proteins with high expression (Supplementary Table 2). These differential proteins were mainly involved in oxidative stress, mitochondrial respiration and energy metabolism, synapse, cytoskeleton, chaperone, and others. Interestingly, two common differential proteins were found in the comparisons. One was isocitrate dehydrogenase [NAD] subunit alpha, mitochondrial (IDH3A), which was reduced in *3 ×Tg* AD mice vs. WT mice but relatively increased after 0.5 mg/kg Xn treatment. The other was protein-L-isoaspartate(D-aspartate) O-methyltransferase, which was down-regulated.

Gene ontology (GO) enrichment analysis was performed to categorize the differential hippocampal proteins (Supplementary Figure 4). Treatment with Xn increased three cellular components (extracellular exosome, myelin sheath and mitochondrion) and decreased the ability of ubiquitin protein ligase binding in the Molecular Function. The Kyoto Encyclopedia of Genes and Genomes (KEGG) pathway was further analyzed to determine high-level functions and utilities of the biological system from the molecular-level information (Fig. 3). We found 19 meaningful pathways in the *3 ×Tg* AD vs. WT mice comparison and 14 in the *3 ×Tg* AD mice treated with 0.5 mg/kg Xn compared to *3 ×Tg* AD control mice. Moreover, there were 12 common pathways of which IDH3A was involved in four, namely metabolic pathways, carbon metabolism, citrate cycle (TCA cycle) and 2-oxocarboxylic acid metabolism.

**Fig. 3:**
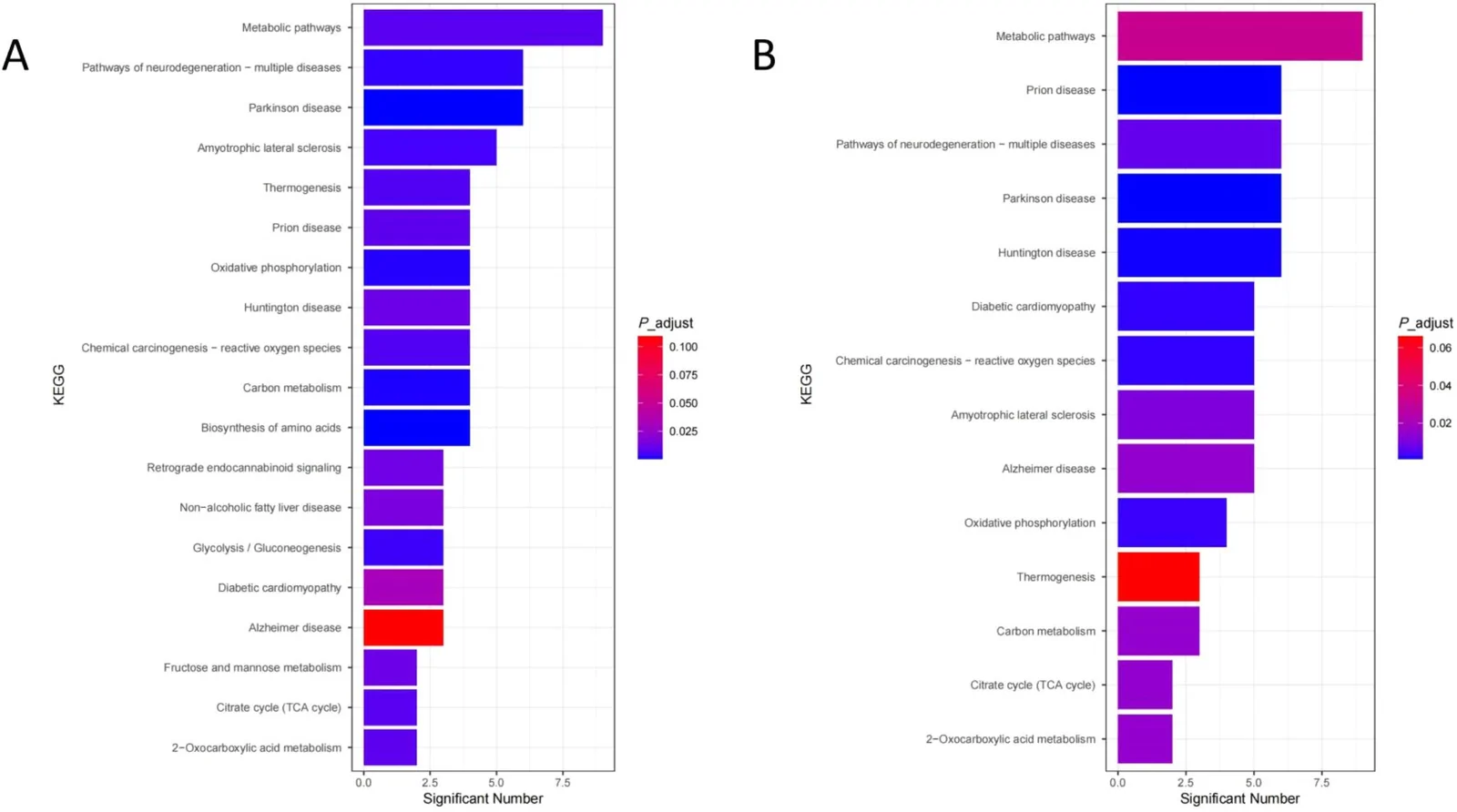
KEGG pathway analysis of differential expressed proteins of hippocampus in 3 ×Tg AD mice. (A) KEGG pathway barplot of 3 ×Tg AD mice compared to WT mice; (B) KEGG pathway barplot of 3 ×Tg AD mice treated with 0.5 mg/kg Xn compared to 3 ×Tg AD control mice.

### 3.4 Xn altered the expression levels of hippocampal proteins in APP/PS1 mice

TMT quantitative proteomics strategy was used to analyze the differential proteins in *APP/PS1* mice treated with Xn. A total of 407 differential hippocampal proteins was detected in *APP/PS1* vs. WT mice, of which 227 proteins were highly expressed and 180 proteins lower expressed. A total of 421 differential proteins were found in *APP/PS1* mice treated with 5 mg/kg Xn versus *APP/PS1* mice, of which 285 proteins were highly expressed and 136 proteins lower expressed. We observed that 5 mg/kg Xn significantly affected the expression levels of hippocampal proteins in AD model mice (Fig. 4). We further found 70 common differential proteins in these two comparisons, of which 39 were increased, 25 were decreased and six remained unchanged after Xn intervention. These common proteins mainly involved the biological functions of binding, catalytic activity, molecular function regulatory activity, structural molecule activity, transporter activity, transcription regulatory activity and others (Supplementary Table 3).

**Fig. 4:**
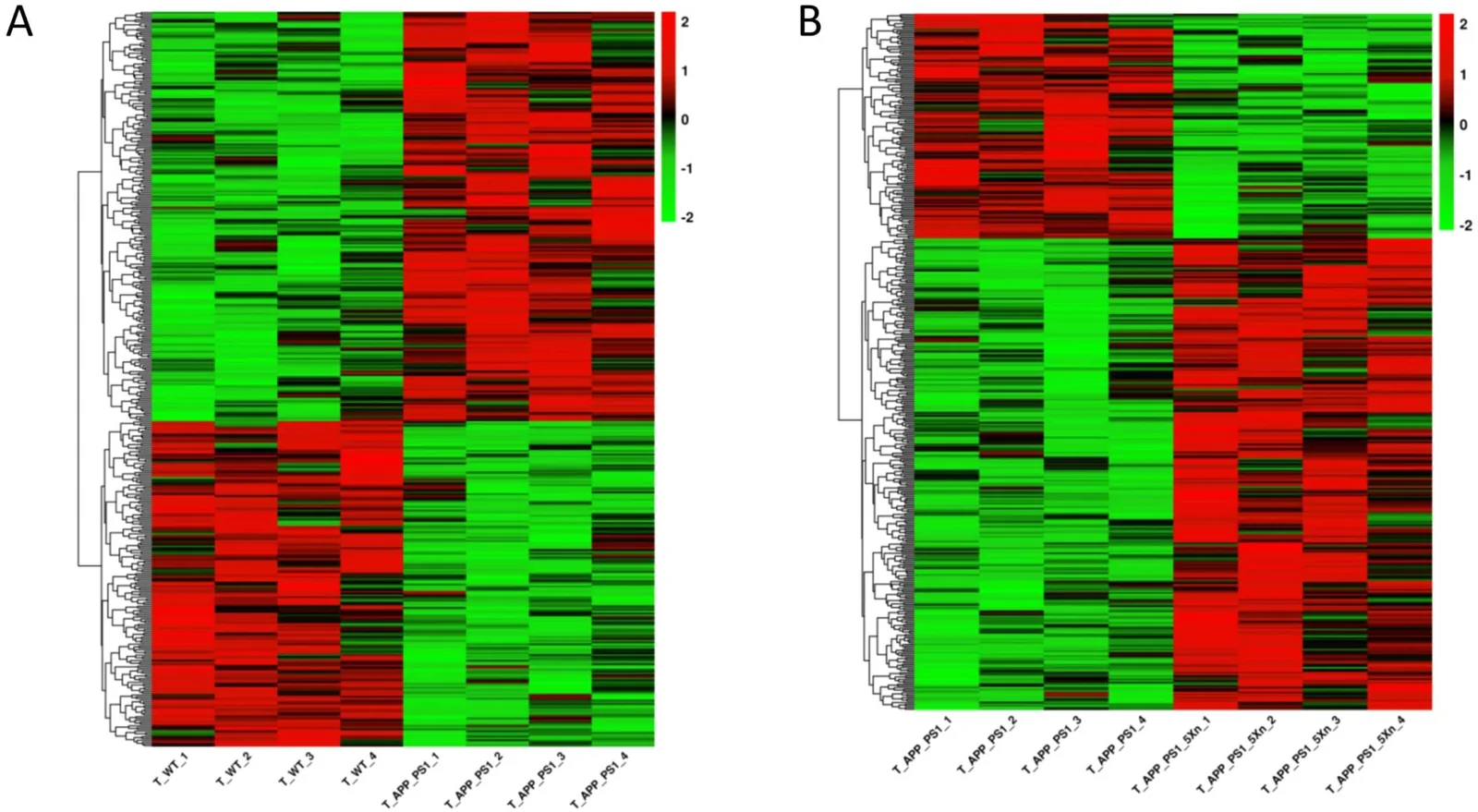
Heat map analysis of significant proteins of hippocampus in different animal groups. (A) and (B) showed the pairwise comparison results for APP/PS1 vs. WT mice and APP/PS1 mice treated with 5 mg/kg Xn vs. Xn-untreated APP/PS1 mice, respectively. Red indicated up-regulated while green indicated down-regulated proteins. The horizontal axis represented samples in the two comparison groups and the vertical axis represented significant proteins. n = 4 per group.

According to DAVID and GO annotation analyses, differential proteins in the hippocampal tissues were classified and enriched according to biological process (BP), cellar component (CC) and molecular function (MF) (Fig. 5). BP was mainly enriched in protein transport, cell cycle, cell division, intracellular protein transport, ion transport and others. CC was mainly enriched in cytoplasm, membrane, cytoskeleton, cytosol, plasma membrane, mitochondrion, nucleoplasm and others. MF was mainly enriched in protein binding, identical protein binding, nucleotide binding, macromolecular complex binding, ATP binding and others. The percent of differential genes in CC and MF was higher than BP in all the comparisons. We further found 42 significant KEGG pathways in a comparison of *APP/PS1* mice versus WT mice, and 46 significant KEGG pathways in a comparison of *APP/PS1* mice with 5 mg/kg Xn versus *APP/PS1* mice (Fig. 6). Moreover, there were 22 common pathways, among which the pathways with more proteins enriched were metabolic pathways, pathways of neurodegeneration-multiple diseases, Alzheimer disease, and others. Interestingly, cytochrome c oxidase subunit 6A1, mitochondrial, ATP synthase F(0) complex subunit B1, mitochondrial, NADH dehydrogenase [ubiquinone] 1 subunit C2 and NADH dehydrogenase [ubiquinone] 1 beta subcomplex subunit 4 were involved in at least ten of these common pathways.

**Fig. 5:**
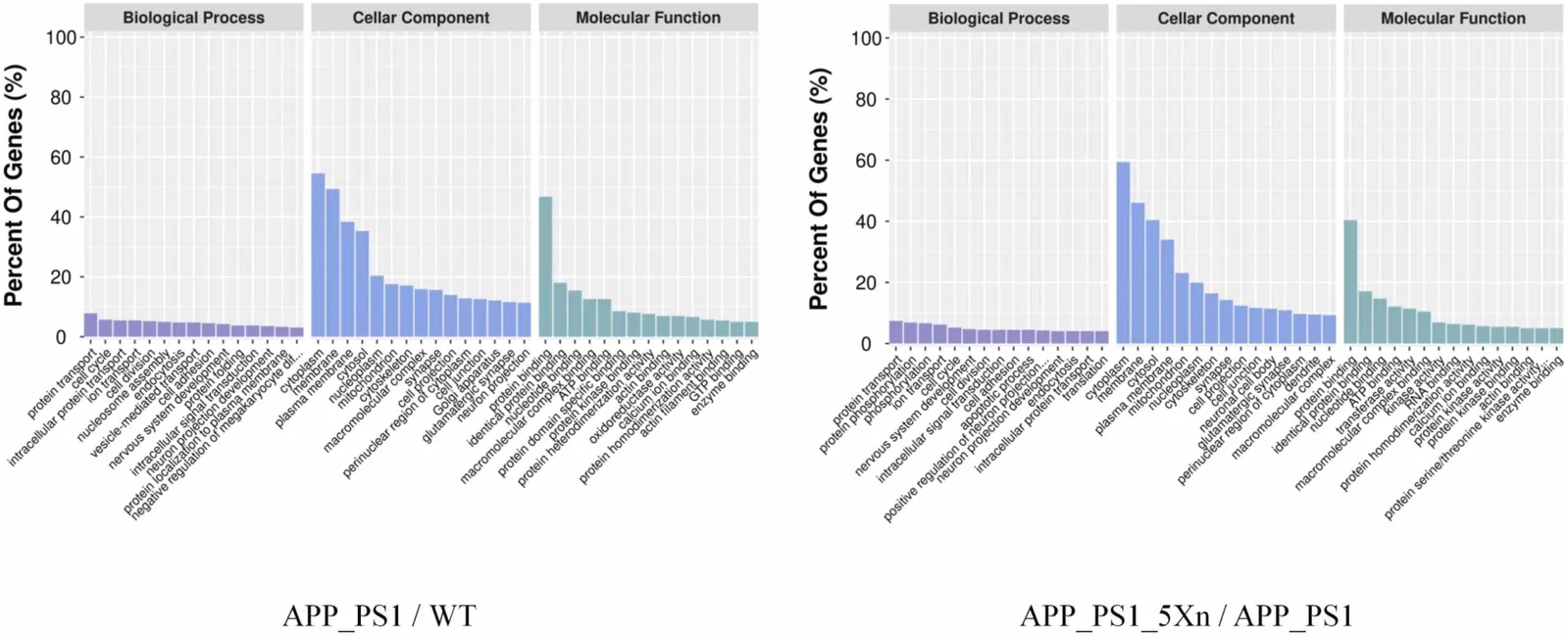
GO enrichment bar plot of the differential genes in different groups. The x-axis describes the GO term. The y-axis represents the percent of differential genes to all genes. The top 15 genes ranked by *P*-value in Biological Process (BP), Cellular Component (CC), and Molecular Function (MF) are shown for APP/PS1 vs. WT mice (left BP, CC & MF series) and Xn-treated APP/PS1 vs. Xn-untreated APP/PS1 mice (right BP, CC & MF series).

**Fig. 6:**
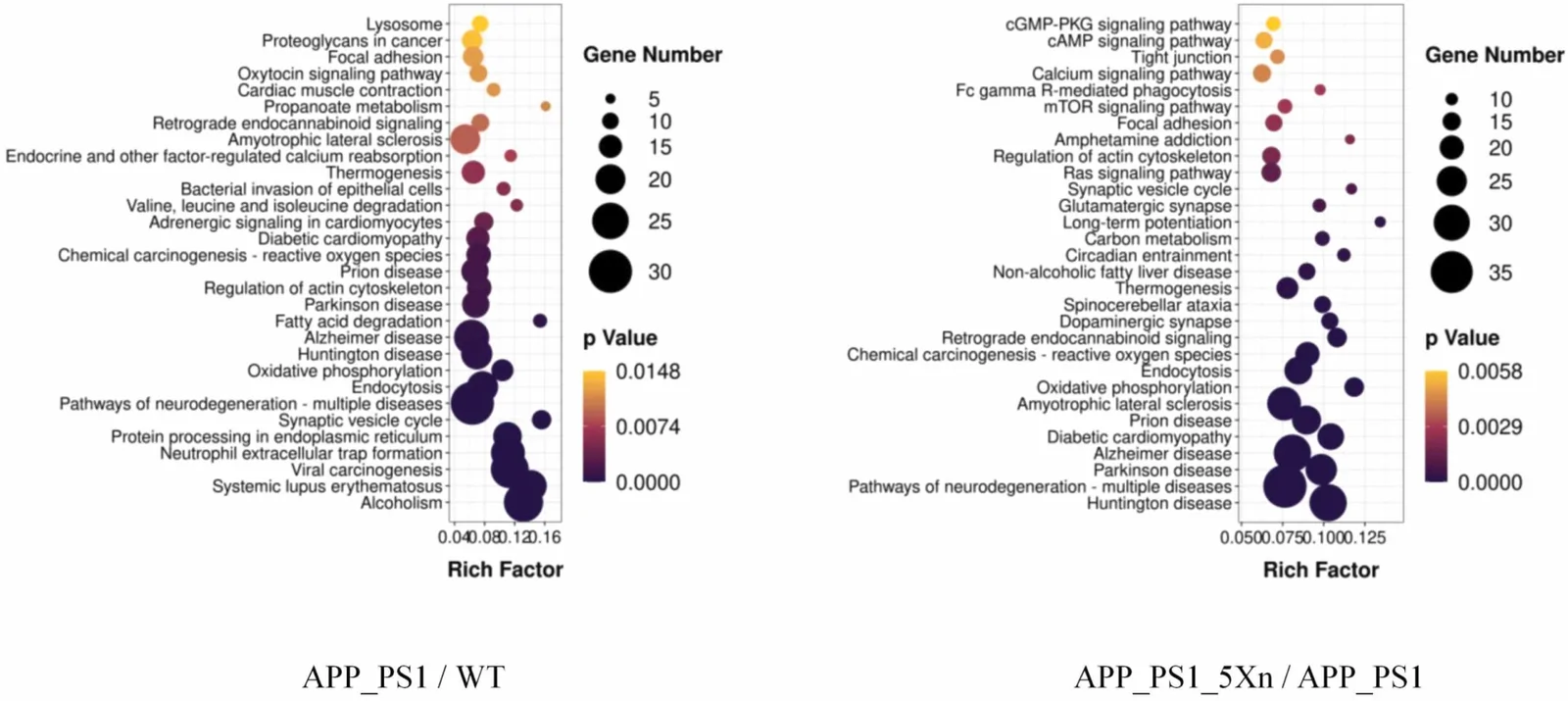
KEGG enrichment scatter plot of the differential genes in different groups. The x-axis is Rich factor, which represents the proportion of significant genes enriched in this pathway relative to background genes. The y-axis is the KEGG pathway name. The figures showed the results of the top 30 pathways ranked by *P*-value. The larger the dot, the more purple the color, indicates more trusted pathways.

### 3.5 Comprehensive analysis of hippocampus proteomics in AD model mice after Xn treatment

To identify potential protein biomarkers and signaling pathways in the hippocampus of AD model mice treated with Xn, we compared and comprehensively analyzed the proteomics results of the two different processing methods (2D-DIGE MALDI-TOF MS/MS and TMT labeling LC-MS/MS technique). Comparison of the KEGG pathway results revealed nine common signaling pathways mainly involved in global metabolism, energy metabolism, neurodegenerative disease, cardiovascular disease and cancer. Specifically, they included: metabolic pathways, pathways of neurodegeneration-multiple diseases, Huntington disease, Amyotrophic lateral sclerosis, Parkinson disease, Prion disease, diabetic cardiomyopathy, chemical carcinogenesis - reactive oxygen species, and oxidative phosphorylation, respectively.

## 4 Discussion

We showed here that systemic Xn treatment significantly ameliorated cognitive dysfunction in two well studied transgenic murine models of AD. Xn had a positive effect on measures of cognitive function of *3 ×Tg* AD mice treated daily i.p. for 6 weeks with 0.5 mg/kg Xn and of *APP/PS1* mice treated every other day by intragastric administration of 5 mg/kg Xn for three months. Xn had been previously reported to improve cognitive impairment (Liu et al., 2023, Paraiso et al., 2020) but rarely in AD model mice (Sun et al., 2021). Here, we reported that Xn treatment significantly improved the ability of stimulating learning and memory and cognitive function of *3 ×Tg* AD mice and ameliorated cognitive decline in *APP/PS1* mice. In sum, Xn treatment significantly ameliorated cognitive dysfunction in two types of AD mice, findings that were consistent with those reported in our previous study (Liu et al., 2022).

We identified molecular changes in the hippocampus of Xn-treated vs. untreated AD mice using well established analytical techniques. 2D-DIGE is the only proteomics method that applies internal standards, which minimizes the systematic error and greatly improves the sensitivity and reliability of the experiment (May et al., 2021, Pasquali et al., 2017). TMT is ideal for analyzing the dose dependence, time course, and biological replication of multiple samples simultaneously (Pagel et al., 2021, Zhang and Elias, 2017). Compared with 2D-DIGE, TMT has the advantage of increasing the relative quantity of quantification, increasing the sample flux, and reducing the missing quantitative information in the samples, so it has a broader application (Guerrero-Castillo et al., 2021). After Xn treatment, we identified 421 differential proteins by TMT in *APP/PS1* mice while only 27 differential proteins were found in *3 ×Tg* AD mice by 2D-DIGE. The number of differential proteins varied by 15–16 times. By comparison, a proteomic analysis performed on yeast cells using TMT labeling and 2-D DIGE identified 842 and 52 proteins, respectively (Westman et al., 2012), which also represented a 16-fold difference in protein number. Nevertheless, unfortunately, we did not find a common differential protein between the two omics approaches.

Proteomics has become a valuable tool in the study of experiments models of AD (Bai et al., 2020, Sung et al., 2023), but such studies are often focused on a single cell, single animal species, or just one analytical technique. Here, we employed 2D-DIGE and TMT labeling technology combined with LC-MS/MS to analyze hippocampal proteomics in two murine AD models. We identified two common differential proteins and 12 signaling pathways in *3 ×Tg* AD mice by 2D-DIGE coupled with MALDI-TOF MS/MS. We further identified 70 common differential proteins and 22 signaling pathways in *APP/PS1* mice by TMT-based LC-MS/MS techniques. Moreover, the combined application of 2D-DIGE and TMT-based proteomics is more conducive to the discovery of disease biomarkers and pathogenesis (Lei et al., 2021, Schulte et al., 2023).

We found nine common signaling pathways which together provide clues for understanding molecular mechanisms that, in the future, will hopefully serve as a foundation for therapeutic approaches to slow or even arrest the clinical course of AD. Systemic treatment with Xn alleviated cognitive impairment in both *3 ×Tg* AD mice and *APP/PS1* mice by regulating the metabolic pathway and the oxidative phosphorylation pathway, which is responsible for generating adenosine triphosphate (ATP). The brain’s continuously high ATP demand renders the organ sensitive to changes in energy fuel supply and mitochondrial function, such that bioenergetic deficits and chronic oxidative stress are both major contributors to AD ((Poddar et al., 2021). Following dysregulation of brain energy metabolism in AD, many cellular repair/regenerative processes are activated to conserve the energy required for cell viability (Preeti et al., 2022, Yu et al., 2022). We previously found Xn could reduce AD-related changes in stably transfected N2a/APP cells by modulating multiple pathogenic pathways, including oxidative stress and others (Huang et al., 2018). Thus, it would appear that an important component of Xn therapeutics in cellular and animal models of AD is the promotion of brain bioenergetics.

The prevention and control of chronic and complex diseases such as AD, diabetes mellitus, cardiovascular disease (CVD), and cancers, will continue to represent critical challenges in human health. Similar to AD, the risk for developing type 2 diabetes mellitus (T2DM) increases with age, and T2DM also increases the risk for AD (Chatterjee and Mudher, 2018). The mechanisms underlying these associations are linked with insulin resistance, neuroinflammation, oxidative stress, mitochondrial dysfunction and metabolic syndrome (Carvalho and Moreira, 2023, Diniz Pereira et al., 2021). Some authors even suggested that AD represents a type 3 diabetes mellitus (T3DM) (Janoutová et al., 2022, Michailidis et al., 2022). Relevant to this hypothesis is the present finding that the metabolic pathways and diabetic cardiomyopathy pathway were both represented among the differential signaling pathways in the hippocampi of the two AD mouse models after Xn treatment. With respect to CVD, prior studies had suggested not only an association with AD progression (Leszek et al., 2021, Zhang et al., 2023) but also a risk factor and potential predictor of AD. Approximately 40% of AD cases was estimated to be preventable by targeting modifiable cardiovascular risk factors (Nordestgaard et al., 2022). Of note, the ApoEƐ4 genotype was found to be one of the major risk factors affecting both the cardiovascular and nervous systems (Saeed et al., 2023). In regard to cancer, epidemiological studies had shown an inverse association between cancer and AD (Lanni et al., 2021, Ren et al., 2022). Two-sample Mendelian randomization (MR) analysis revealed a significant causal influence for increased AD risk on reduced cancer risk (Dong et al., 2023). AD may result in lower cancer risk, which suggested that susceptibility to one disease may protect against the other. Therefore, the results of our proteomic analysis may be useful in elucidating these potential relationships at a molecular level.

Although the present study identifies potential protein markers and KEGG pathways involved in the ameliorative action of Xn on cognitive decline in mouse models of AD, significant shortcomings should be recognized. Firstly, although we conducted a comparison between two proteomic experiments, the types of AD model mice, the routes of Xn administration and the techniques of proteomic analysis, all differ one from the other. The more reliable approach it might be argued would be to employ singular approaches. On the other hand, results from the present methodological design might be considered solid because of the overlapping findings from the multiple approaches employed. Secondly, hippocampal proteomic analysis was only performed on groups of mice that showed clear-cut behavioral results, not for all groups, which may have resulted in missed differential information. Thirdly, the screened protein markers or key signaling pathways were not further verified. In addition, more AD-related characteristic indicators such as senile plaques and Tau proteins were not shown except for the behavioral data. Finally, while the highest doses of Xn treatment yielded significantly positive behavioral and brain proteomic data, the present study was not designed to test for a dose-response relationship. However, on the positive side, Xn treatment appeared to slow the course of cognitive decline in the absence of any apparent adverse side effects. Additionally, a surprising result from the MMW test was that Xn appeared to speed the temporal acquisition of memory in normal (WT) young adult mice as shown by the significantly greater reduction of escape latency over the 5-day test period in Xn-treated vs. -untreated animals. If confirmed in future studies, this might indicate a role for Xn in both cognitive development as well cognitive decline.

## 5 Conclusions

Based on the positive cognitive outcomes of Xn treatment in paired behavioral studies of two mouse models of AD, we compared two proteomic techniques to identify potential protein targets and signaling pathways in brain hippocampi. The results provided new insight into the molecular mechanisms underlying the murine neuroprotective effects of Xn, and the use of this natural, hop-derived prenylated chalcone (3′-(3,3-dimethylallyl)-2′,4′,4-trihydroxy-6′-methoxychalcone) for food additives or drug research and development primarily in relation to AD.

## Statement of significance

Integrating 2D-DIGE and TMT-based quantitative proteomics in two Alzheimer's disease (AD) mouse models (3 ×Tg and APP/PS1), this study demonstrates that xanthohumol (Xn) improves cognitive function and ameliorates cerebral energy metabolism deficits by modulating core oxidative phosphorylation and metabolic networks. By linking AD pathology to pathways involved in cardiovascular disease, diabetes, and cancer, these findings significantly expand our understanding of Xn's multi-targeted neuroprotective mechanisms and provide crucial molecular evidence for natural product-based AD therapeutics.

## CRediT authorship contribution statement

**Wei Liu:** Writing – review & editing, Writing – original draft, Visualization, Software, Methodology, Investigation, Funding acquisition, Formal analysis, Data curation, Conceptualization. **Benhong Xu:** Writing – review & editing, Software, Methodology, Formal analysis. **Chen Yang:** Software, Resources, Investigation. **Guowei Li:** Resources, Investigation. **Xifei Yang:** Writing – review & editing, Project administration, Funding acquisition, Conceptualization.

## Ethics declaration

This study was conducted in accordance with the following guidelines for animal welfare and/or reporting: the Declaration of Helsinki. This study was approved by the the ethics committee of Shenzhen Center for Disease Control and Prevention. (Approval No. No. 2019034, No. 2022019)

## Consent to Publish declaration

Not applicable.

## Funding

This work was supported by Shenzhen Basic Research Project of Science and Technology Plan (JCYJ20200109143431341), Key Basic Research Program of Shenzhen Science and Technology Innovation Commission (JCYJ20200109150717745), and Guangdong Project of Medical Science and Technology Research (A2023365), and Shenzhen Key Medical Discipline Construction Fund (SZXK069) and 10.13039/501100012151Sanming Project of Medicine in Shenzhen (SZSM202211010). The funding agencies played no role in the design, collection, analysis or interpretation of data reported in this paper.

## Clinical trial number

Not applicable.

## Declaration of Competing Interest

The authors declare that they have no known competing financial interests or personal relationships that could have appeared to influence the work reported in this paper.

## Data Availability

The data presented in this study were available on request from the corresponding author.
