# Supplementary material for: Xanthohumol promotes cognitive function-associated neuroprotein changes in two mouse models of Alzheimer’s disease
Source: IBRO Neurosci Rep. 2026 Oct 23;21:1113–21. doi: 10.1016/j.ibneur.2026.10.013 (PMC13755968; doi:10.1016/j.ibneur.2026.10.013)
Supplement: Supplementary file 1 [file mmc1.docx]

**Supplementary Materials**

**Xanthohumol promotes cognitive function-associated neuroprotein changes in two mouse models of Alzheimer’s disease**

**Contents**

Supplementary Figure 1 Schematic diagram of the experimental design.

Supplementary Figure 2 Xn ameliorated learning and memory deficits in APP/PS1 mice.

Supplementary Figure 3 Identified differential protein spots on 2D-DIGE image of hippocampus proteins.

Supplementary Figure 4 Gene Ontology enrichment analysis of differentially expressed proteins in the hippocampus.

Supplementary Table 1 Identification of hippocampal differential expressed proteins in *3×Tg* AD mice vs. WT mice.

Supplementary Table 2 Identification results of differentially expressed hippocampal proteins in *3×Tg* AD mice treated with 0.5mg/kg Xn compared with *3×Tg* AD control mice.

Supplementary Table 3 Seventy common differential proteins of hippocampus identified by TMT quantitative proteomic techniques in *APP/PS1* mice treated with or without 0.5mg/kg Xn compared to control mice.

Supplementary Figure 1 Schematic diagram of the experimental design.

Supplementary Figure 2 Xn ameliorated learning and memory deficits in APP/PS1 mice.

(A) escape latency during platform trial test. For each group of mice, spatial learning improved significantly with increasing training time. Xn-treated animals showed better spatial learning ability compared with untreated animals; (B) average speed during the probe test; (C) representative swimming trajectory graphs; (D) number of crossing movements; (E) percentage of distance traveled in the target quadrant; (F) percentage of time spent in the target quadrant. n=11-14 per group (seven male and six female in WT group; six male and five female in WT with 5 mg/kg Xn group; seven male and seven female in APP/PS1 with or without 5 mg/kg Xn group). * *P*<0.05, ** *P*<0.01, *** *P*<0.001. Error bar, standard error of mean (SEM). Note that Figures A, D, and F reported in our previous work (Liu W, et al. 2022) and the lower two trajectory graphs in C published in our previous study (Liu W, et al. 2024).

Supplementary Figure 3 Identified differential protein spots on 2D-DIGE image of hippocampus proteins.

(A) *3×Tg* AD mice compared to WT mice. (B) *3×Tg* AD mice treated with 0.5mg/kg Xn compared to *3×Tg* AD control mice. Vertical from top to bottom indicates the molecular weight from large to small. Horizontal from left to right indicates pH 3 to 11.

Supplementary Figure 4 Gene Ontology enrichment analysis of differentially expressed proteins in the hippocampus.

(A-C) comparison results of 3×Tg AD mice vs. WT mice; (D-F) comparison results of 3×Tg AD mice treated with 0.5mg/kg Xn vs. 3×Tg AD control mice. (A), (D) biological process; (B), (E) cellular components; (C), (F) molecular function. n=6 per group.

Supplementary Table 1 Identification of hippocampal differential expressed proteins in *3×Tg* AD mice vs. WT mice.

| Spot No. | Accession No. | Protein name | Uniport | Mascot score | MW(Da) | pI-Value | 3×Tg vs. WT | |
| --- | --- | --- | --- | --- | --- | --- | --- | --- |
|  |  |  |  |  |  |  | *P*-Value | Ratio |
| Oxidative stress | | | | | | | | |
| 23 | IDH3A_MOUSE | Isocitrate dehydrogenase [NAD] subunit alpha, mitochondrial | Q9D6R2 | 467 | 40069 | 6.3 | 0.028 | -1.06 |
| 13 | SODC_MOUSE | Superoxide dismutase [Cu-Zn] | P08228 | 691 | 16104 | 6 | 0.017 | -1.09 |
| 8 | IDH3A_MOUSE | Isocitrate dehydrogenase [NAD] subunit alpha, mitochondrial | Q9D6R2 | 546 | 40069 | 6.3 | 0.0093 | -1.09 |
| 19 | QCR7_MOUSE | Cytochrome b-c1 complex subunit 7 | Q9D855 | 130 | 13519 | 9.5 | 0.027 | 1.42 |
| Mitochondrial respiration and metabolism | | | | | | | | |
| 6 | NDUV2_MOUSE | NADH dehydrogenase [ubiquinone] flavoprotein 2, mitochondrial | Q9D6J6 | 262 | 27610 | 7.8 | 0.0036 | -1.31 |
| 15 | NDUS5_MOUSE | NADH dehydrogenase [ubiquinone] iron-sulfur protein | Q99LY9 | 451 | 12867 | 10 | 0.021 | 1.72 |
| 9 | KAD1_MOUSE | Adenylate kinase isoenzyme 1 | Q9R0Y5 | 323 | 21640 | 5.6 | 0.0095 | -1.14 |
| 24 | NDUAA_MOUSE | NADH dehydrogenase [ubiquinone] 1 alpha subcomplex subunit 1 | Q99LC3 | 95 | 40863 | 8.5 | 0.032 | -1.09 |
| Synaptic related proteins | | | | | | | | |
| 5 | GLNA_MOUSE | Glutamine synthetase | P15105 | 141 | 42834 | 6.7 | 0.0027 | -1.17 |
| 17 | SYN2_MOUSE | Synapsin-2 | Q64332 | 50 | 63618 | 9.3 | 0.023 | 1.28 |
| 32 | GLNA_MOUSE | Glutamine synthetase | P15105 | 141 | 42834 | 6.7 | 0.039 | -1.07 |
| 36 | SYN2_MOUSE | Synapsin-2 | Q64332 | 126 | 63618 | 9.3 | 0.05 | -1.17 |
| Cytoskeletal proteins | | | | | | | | |
| 11 | SEPT5_MOUSE | Septin-5 | Q9Z2Q6 | 295 | 43177 | 6.2 | 0.012 | -1.17 |
| 29 | SEP11_MOUSE | Septin-11 | Q8C1B7 | 173 | 50005 | 6.2 | 0.036 | -1.12 |
| 18 | TMOD2_MOUSE | Tropomodulin-2 | Q9JKK7 | 377 | 39487 | 5.1 | 0.026 | 1.21 |
| 7 | ACTB_MOUSE | Actin, cytoplasmic 1 | P60710 | 438 | 42052 | 5.2 | 0.0057 | 1.22 |
| 10 | ACTB_MOUSE | Actin, cytoplasmic 1 | P60710 | 419 | 42052 | 5.2 | 0.011 | 1.2 |
| 25 | ACTB_MOUSE | Actin, cytoplasmic 1 | P60710 | 375 | 42052 | 5.2 | 0.032 | 1.29 |
| 26 | ACTB_MOUSE | Actin, cytoplasmic 1 | P60710 | 318 | 42052 | 5.2 | 0.033 | 1.25 |
| 34 | ALDOC_MOUSE | Fructose-bisphosphate aldolase C | P05063 | 472 | 39769 | 6.8 | 0.044 | -1.13 |
| Chaperone | | | | | | | | |
| 33 | CH60_MOUSE | 60 kDa heat shock protein, mitochondrial | P63038 | 523 | 61088 | 5.8 | 0.043 | -1.19 |
| 20 | TCPB_MOUSE | T-complex protein 1 subunit beta | P80314 | 292 | 57783 | 6 | 0.027 | -1.08 |
| 37 | CH60_MOUSE | 60 kDa heat shock protein, mitochondrial | P63038 | 178 | 61088 | 5.8 | 0.023 | -1.08 |
| Others | | | | | | | | |
| 2 | PIMT_MOUSE | Protein-L-isoaspartate(D-aspartate) O-methyltransferase | P23506 | 164 | 24676 | 7.9 | 0.00098 | -1.12 |
| 4 | UBE2N_MOUSE | Ubiquitin-conjugating enzyme E2 N | P61089 | 196 | 17184 | 6.2 | 0.0027 | 2.1 |
| 21 | ODP2_MOUSE | Dihydrolipoyllysine-residue acetyltransferase component of p | Q8BMF4 | 152 | 68469 | 9.6 | 0.027 | 1.11 |
| 22 | UB2L3_MOUSE | Ubiquitin-conjugating enzyme E2 L3 | P68037 | 65 | 18021 | 9.5 | 0.028 | -1.08 |
| 28 | TPIS_MOUSE | Triosephosphate isomerase | P17751 | 373 | 32684 | 5.5 | 0.036 | -1.08 |
| 35 | IPYR_MOUSE | Inorganic pyrophosphatase | Q9D819 | 83 | 33102 | 5.3 | 0.05 | -1.08 |
| 38 | FKB1A_MOUSE | Peptidyl-prolyl cis-trans isomerase FKBP1A | P26883 | 244 | 11972 | 9.1 | 0.047 | 1.54 |

Supplementary Table 2 Identification results of differentially expressed hippocampal proteins in *3×Tg* AD mice treated with 0.5mg/kg Xn compared with *3×Tg* AD control mice.

| Spot No. | Accession No. | Protein name | Mascot score | Coverage(%) | MW(Da) | pI-Value | 3×Tg 0.5Xn vs. 3×Tg | |
| --- | --- | --- | --- | --- | --- | --- | --- | --- |
|  |  |  |  |  |  |  | *P*-Value | Ratio |
| Oxidative stress | | | | | | | | |
| 23 | IDH3A_MOUSE | Isocitrate dehydrogenase [NAD] subunit alpha, mitochondrial | Q9D6R2 | 467 | 40069 | 6.3 | 0.014 | 1.08 |
| 104 | QCR1_MOUSE | Cytochrome b-c1 complex subunit 1, mitochondrial | Q9CZ13 | 358 | 53446 | 5.8 | 0.039 | 1.85 |
| 45 | UCHL1_MOUSE | Ubiquitin carboxyl-terminal hydrolase isozyme L1 | Q9R0P9 | 377 | 25165 | 5 | 0.005 | 1.08 |
| 89 | SODM_MOUSE | Superoxide dismutase [Mn], mitochondrial | P09671 | 120 | 24816 | 9.5 | 0.024 | 1.12 |
| 87 | ODPA_MOUSE | Pyruvate dehydrogenase E1 component subunit alpha, somatic f | P35486 | 135 | 43888 | 9.4 | 0.016 | 1.14 |
| 81 | UCHL1_MOUSE | Ubiquitin carboxyl-terminal hydrolase isozyme L1 | Q9R0P9 | 296 | 25165 | 5 | 0.036 | 1.35 |
| Mitochondrial respiration and energy metabolism | | | | | | | | |
| 56 | GLOD4_MOUSE | Glyoxalase domain-containing protein 4 | Q9CPV4 | 359 | 33581 | 5.2 | 0.046 | -1.18 |
| 47 | ATPB_MOUSE | ATP synthase subunit beta, mitochondrial | P56480 | 664 | 56265 | 5.1 | 0.017 | -1.11 |
| 98 | LGUL_MOUSE | Lactoylglutathione lyase | Q9CPU0 | 200 | 20967 | 5.1 | 0.0029 | 1.86 |
| 74 | ATPA_MOUSE | ATP synthase subunit alpha, mitochondrial | Q03265 | 404 | 59830 | 9.7 | 0.036 | -1.1 |
| Synaptic related proteins | | | | | | | | |
| 41 | CALM_MOUSE | Calmodulin | P62204 | 131 | 16827 | 3.9 | 0.00099 | -1.66 |
| 55 | SH3G2_MOUSE | Endophilin-A1 | Q62420 | 266 | 40101 | 5.1 | 0.045 | -1.11 |
| 44 | CPLX2_MOUSE | Complexin-2 | P84086 | 95 | 15499 | 4.9 | 0.0016 | 1.31 |
| 49 | DPYL2_MOUSE | Dihydropyrimidinase-related protein 2 | O08553 | 383 | 62638 | 5.9 | 0.021 | -1.19 |
| Cytoskeletal proteins | | | | | | | | |
| 42 | ALDOA_MOUSE | Fructose-bisphosphate aldolase A | P05064 | 290 | 39787 | 9.2 | 0.001 | 1.14 |
| 52 | COF2_MOUSE | Cofilin-2 | P45591 | 194 | 18812 | 8.8 | 0.035 | 1.62 |
| 48 | ALDOA_MOUSE | Fructose-bisphosphate aldolase A | P05064 | 164 | 39787 | 9.2 | 0.02 | 1.07 |
| Chaperone | | | | | | | | |
| 50 | HSP7C_MOUSE | Heat shock cognate 71 kDa protein | P63017 | 56 | 71055 | 5.2 | 0.0086 | 1.49 |
| Others | | | | | | | | |
| 108 | GUAD_MOUSE | Guanine deaminase | Q9R111 | 123 | 51494 | 5.3 | 0.047 | 1.15 |
| 31 | GRP75_MOUSE | Stress-70 protein, mitochondrial | P38647 | 272 | 73768 | 5.8 | 0.0029 | 1.49 |
| 53 | HINT1_MOUSE | Histidine triad nucleotide-binding protein 1 | P70349 | 197 | 13882 | 6.4 | 0.035 | 1.08 |
| 96 | PSMD9_MOUSE | 26S proteasome non-ATPase regulatory subunit 9 | Q9CR00 | 38 | 24876 | 6 | 0.00026 | -1.15 |
| 2 | PIMT_MOUSE | Protein-L-isoaspartate(D-aspartate) O-methyltransferase | P23506 | 164 | 24676 | 7.9 | 0.0072 | -1.08 |
| 99 | VATA_MOUSE | V-type proton ATPase catalytic subunit A | P50516 | 432 | 68625 | 5.3 | 0.0083 | 1.18 |
| 94 | VDAC1_MOUSE | Voltage-dependent anion-selective channel protein 1 | Q60932 | 189 | 32502 | 9.2 | 0.012 | -1.11 |
| 80 | SNAG_MOUSE | Gamma-soluble NSF attachment protein | Q9CWZ7 | 385 | 35052 | 5.2 | 0.017 | -1.2 |
| 93 | GBB1_MOUSE | Guanine nucleotide-binding protein G(I)/G(S)/G(T) subunit be | P62874 | 210 | 38151 | 5.6 | 0.04 | -1.13 |

Supplementary Table 3 Seventy common differential proteins of hippocampus identified by TMT quantitative proteomic techniques in APP/PS1 mice treated with or without 0.5mg/kg Xn compared to control mice.

| Accession ID | Protein Name | Score | Coverage | Unique Peptides | APP/PS1 vs. WT | | APP/PS1 5Xn vs. APP/PS1 | |
| --- | --- | --- | --- | --- | --- | --- | --- | --- |
|  |  |  |  |  | *P*-value | Fold | *P*-value | Fold |
| Binding | | | | | | | | |
| Q7TQH0 | Ataxin-2-like protein | 69.98 | 16.78 | 13 | 0.0004 | 0.97 | 0.0101 | 1.05 |
| Q5DQR4 | Syntaxin-binding protein 5-like | 157.48 | 27.26 | 23 | 0.0464 | 0.97 | 0.0216 | 1.05 |
| Q60780 | Growth arrest-specific protein 7 | 77.90 | 32.07 | 11 | 0.0088 | 0.93 | 0.0235 | 1.06 |
| Q6PER3 | Microtubule-associated protein RP/EB family member 3 | 107.31 | 49.11 | 12 | 0.0013 | 0.95 | 0.0187 | 1.03 |
| Q7TQD2 | Tubulin polymerization-promoting protein | 256.49 | 64.22 | 15 | 0.0203 | 0.94 | 0.0437 | 1.05 |
| Q6ZPQ6 | Membrane-associated phosphatidylinositol transfer protein 2 | 95.92 | 16.93 | 18 | 0.0463 | 0.98 | 0.0071 | 1.03 |
| Q8BIZ1 | Ankyrin repeat and sterile alpha motif domain-containing protein 1B | 150.82 | 14.14 | 16 | 0.0148 | 0.96 | 0.0103 | 1.05 |
| Q8BMI3 | ADP-ribosylation factor-binding protein GGA3 | 52.51 | 19.50 | 11 | 0.0100 | 0.93 | 0.0398 | 1.08 |
| Q8QZT2 | Centriole, cilia and spindle-associated protein | 51.76 | 38.89 | 7 | 0.0071 | 0.93 | 0.0275 | 1.05 |
| Q8VD63 | Testis-specific Y-encoded-like protein 4 | 16.79 | 10.59 | 4 | 0.0344 | 0.94 | 0.0306 | 1.07 |
| Q9D7S7 | 60S ribosomal protein L22-like 1 | 7.51 | 15.57 | 2 | 0.0426 | 0.96 | 0.0014 | 1.11 |
| Q9JKK1 | Syntaxin-6 | 20.22 | 25.88 | 4 | 0.0071 | 0.94 | 0.0167 | 1.05 |
| Q9JMC3 | DnaJ homolog subfamily A member 4 | 14.47 | 10.83 | 4 | 0.0499 | 0.96 | 0.0180 | 1.06 |
| Q9QXS6 | Drebrin | 379.63 | 52.97 | 28 | 0.0241 | 0.97 | 0.0336 | 1.03 |
| Q9WV55 | Vesicle-associated membrane protein-associated protein A | 91.30 | 49.80 | 12 | 0.0338 | 0.96 | 0.0353 | 1.02 |
| Q8VH51 | RNA-binding protein 39 | 37.91 | 11.51 | 5 | 0.0217 | 1.06 | 0.0103 | 0.93 |
| P0C0S6 | Histone H2A.Z | 68.07 | 53.91 | 3 | 0.0261 | 1.12 | 0.0428 | 0.94 |
| P80317 | T-complex protein 1 subunit zeta | 259.54 | 56.50 | 26 | 0.0152 | 1.02 | 0.0344 | 0.98 |
| Q61001 | Laminin subunit alpha-5 | 97.61 | 7.69 | 18 | 0.0125 | 1.11 | 0.0084 | 0.91 |
| Q61739 | Integrin alpha-6 | 14.59 | 6.51 | 4 | 0.0351 | 1.11 | 0.0337 | 0.88 |
| Q6PDL0 | Cytoplasmic dynein 1 light intermediate chain 2 | 130.42 | 30.89 | 13 | 0.0031 | 1.04 | 0.0187 | 0.98 |
| Q8BFZ9 | Erlin-2 | 92.92 | 33.24 | 9 | 0.0238 | 1.08 | 0.0049 | 0.96 |
| Q8CCK0 | Core histone macro-H2A.2 | 87.14 | 44.35 | 10 | 0.0313 | 1.06 | 0.0206 | 0.93 |
| Q91ZM2 | SH2B adapter protein 1 | 33.21 | 13.36 | 6 | 0.0281 | 1.13 | 0.0110 | 0.91 |
| Q9EQP2 | EH domain-containing protein 4 | 88.07 | 31.24 | 11 | 0.0014 | 1.10 | 0.0299 | 0.96 |
| Q9QUI0 | Transforming protein RhoA | 135.53 | 60.62 | 6 | 0.0404 | 1.04 | 0.0424 | 0.96 |
| Q9WTR5 | Cadherin-13 | 81.97 | 18.21 | 8 | 0.0487 | 1.08 | 0.0091 | 0.87 |
| Q9WVA4 | Transgelin-2 | 36.96 | 36.18 | 4 | 0.0105 | 1.19 | 0.0226 | 0.86 |
| O89051 | Integral membrane protein 2B | 40.06 | 27.82 | 7 | 0.0102 | 1.09 | 0.0033 | 1.08 |
| Catalytic activity | | | | | | | | |
| P17183 | Gamma-enolase | 760.40 | 87.56 | 30 | 0.0489 | 1.02 | 0.0054 | 0.96 |
| P51174 | Long-chain specific acyl-CoA dehydrogenase, mitochondrial | 147.78 | 36.98 | 14 | 0.0113 | 1.05 | 0.0214 | 0.94 |
| Q8BFP9 | [Pyruvate dehydrogenase (acetyl-transferring)] kinase isozyme 1, mitochondrial | 64.11 | 34.10 | 10 | 0.0072 | 1.08 | 0.0311 | 0.93 |
| Q9EQ20 | Methylmalonate-semialdehyde dehydrogenase [acylating], mitochondrial | 272.59 | 57.01 | 27 | 0.0173 | 1.04 | 0.0135 | 0.96 |
| Q9R0N0 | Galactokinase | 27.23 | 20.15 | 6 | 0.0388 | 1.06 | 0.0054 | 0.95 |
| Q6A4J8 | Ubiquitin carboxyl-terminal hydrolase 7 | 140.51 | 28.56 | 26 | 0.0187 | 0.97 | 0.0041 | 1.04 |
| Q7TME0 | Phospholipid phosphatase-related protein type 4 | 110.15 | 31.20 | 17 | 0.0345 | 0.96 | 0.0056 | 1.06 |
| Q80TA6 | Myotubularin-related protein 12 | 19.26 | 9.64 | 5 | 0.0453 | 0.95 | 0.0074 | 1.05 |
| Q8BUV3 | Gephyrin | 234.87 | 45.12 | 31 | 0.0406 | 0.96 | 0.0031 | 1.05 |
| P97470 | Serine/threonine-protein phosphatase 4 catalytic subunit | 19.15 | 12.38 | 2 | 0.0034 | 1.07 | 0.0424 | 1.05 |
| Q00612 | Glucose-6-phosphate 1-dehydrogenase X | 73.32 | 25.44 | 11 | 0.0384 | 1.03 | 0.0463 | 1.02 |
| Q91YI0 | Argininosuccinate lyase | 63.10 | 22.20 | 11 | 0.0087 | 1.04 | 0.0226 | 1.08 |
| Molecular function regulator activity | | | | | | | | |
| P43024 | Cytochrome c oxidase subunit 6A1, mitochondrial | 51.48 | 61.26 | 4 | 0.0188 | 1.08 | 0.0106 | 0.94 |
| Q9Z239 | Phospholemman | 4.20 | 13.04 | 1 | 0.0059 | 1.39 | 0.0128 | 0.82 |
| Q925E7 | Serine/threonine-protein phosphatase 2A 55 kDa regulatory subunit B delta isoform | 81.19 | 28.26 | 4 | 0.0046 | 0.94 | 0.0210 | 1.04 |
| Structural molecule activity | | | | | | | | |
| Q6PH08 | ERC protein 2 | 346.09 | 47.96 | 39 | 0.0048 | 0.96 | 0.0067 | 1.04 |
| Q9QYX7 | Protein piccolo | 927.11 | 31.81 | 124 | 0.0084 | 0.97 | 0.0012 | 1.05 |
| Transporter activity | | | | | | | | |
| Q3TXX4 | Vesicular glutamate transporter 1 | 70.45 | 16.79 | 4 | 0.0105 | 0.94 | 0.0320 | 1.04 |
| Q8K596 | Sodium/calcium exchanger 2 | 205.30 | 29.97 | 19 | 0.0074 | 0.94 | 0.0008 | 1.06 |
| Transcription regulator activity | | | | | | | | |
| P59759 | MKL/myocardin-like protein 2 | 49.15 | 17.13 | 12 | 0.0165 | 0.96 | 0.0131 | 1.04 |
| Others | | | | | | | | |
| O35737 | Heterogeneous nuclear ribonucleoprotein H | 151.35 | 42.32 | 6 | 0.0409 | 1.03 | 0.0105 | 0.95 |
| P97927 | Laminin subunit alpha-4 | 16.22 | 3.30 | 5 | 0.0023 | 1.11 | 0.0070 | 0.94 |
| Q63829 | COMM domain-containing protein 3 | 16.71 | 34.36 | 5 | 0.0245 | 1.12 | 0.0164 | 0.91 |
| Q8BWT1 | 3-ketoacyl-CoA thiolase, mitochondrial | 210.27 | 70.78 | 20 | 0.0280 | 1.08 | 0.0362 | 0.94 |
| Q6PDH0 | Pleckstrin homology-like domain family B member 1 | 9.38 | 2.04 | 2 | 0.0081 | 1.21 | 0.0377 | 0.88 |
| P08226 | Apolipoprotein E | 170.59 | 67.20 | 20 | 0.0001 | 1.19 | 0.0028 | 1.05 |
| Q9CQQ7 | ATP synthase F(0) complex subunit B1, mitochondrial | 104.50 | 41.02 | 11 | 0.0012 | 1.04 | 0.0085 | 1.04 |
| Q07409 | Contactin-3 | 7.24 | 3.02 | 2 | 0.0157 | 0.93 | 0.0422 | 1.08 |
| Q3UNH4 | G protein-regulated inducer of neurite outgrowth 1 | 361.26 | 59.87 | 45 | 0.0323 | 0.96 | 0.0006 | 1.05 |
| Q56A07 | Sodium channel subunit beta-2 | 71.48 | 38.14 | 9 | 0.0044 | 0.94 | 0.0392 | 1.03 |
| Q80XA6 | RalBP1-associated Eps domain-containing protein 2 | 45.99 | 19.77 | 7 | 0.0002 | 0.93 | 0.0027 | 1.06 |
| Q8BKX1 | Brain-specific angiogenesis inhibitor 1-associated protein 2 | 226.49 | 55.89 | 28 | 0.0030 | 0.96 | 0.0130 | 1.03 |
| Q8CJ19 | [F-actin]-monooxygenase MICAL3 | 98.88 | 13.50 | 22 | 0.0127 | 0.97 | 0.0039 | 1.06 |
| Q8R3V5 | Endophilin-B2 | 101.18 | 49.75 | 16 | 0.0008 | 0.94 | 0.0010 | 1.04 |
| Q91VN4 | MICOS complex subunit Mic25 | 91.96 | 40.66 | 10 | 0.0247 | 0.97 | 0.0209 | 1.05 |
| Q91XV3 | Brain acid soluble protein 1 | 539.24 | 85.84 | 21 | 0.0011 | 0.95 | 0.0237 | 1.04 |
| Q9CQ54 | NADH dehydrogenase [ubiquinone] 1 subunit C2 | 56.27 | 37.50 | 5 | 0.0109 | 0.97 | 0.0148 | 1.03 |
| Q9CQC7 | NADH dehydrogenase [ubiquinone] 1 beta subcomplex subunit 4 | 80.33 | 54.26 | 8 | 0.0072 | 0.95 | 0.0179 | 1.03 |
| Q9CY18 | Sorting nexin-7 | 5.98 | 6.20 | 2 | 0.0208 | 0.92 | 0.0214 | 1.07 |
| Q9JK48 | Endophilin-B1 | 48.51 | 22.74 | 7 | 0.0352 | 0.97 | 0.0005 | 1.05 |
| Q9Z0R6 | Intersectin-2 | 51.59 | 8.44 | 8 | 0.0260 | 0.95 | 0.0018 | 1.06 |
